# Comparison of four international cardiovascular disease prediction models and the prevalence of eligibility for lipid lowering therapy in HIV infected patients on antiretroviral therapy

**DOI:** 10.3325/cmj.2015.56.14

**Published:** 2015-02

**Authors:** Josip Begovac, Gordana Dragović, Klaudija Višković, Jovana Kušić, Marta Perović Mihanović, Davorka Lukas, Đorđe Jevtović

**Affiliations:** 1 University Hospital for Infectious Diseases, Zagreb, Croatia; 2 Department of Infectious Diseases, University of Zagreb School of Medicine, Zagreb, Croatia; 3 Department of Pharmacology, Clinical Pharmacology and Toxicology School of Medicine, University of Belgrade, Belgrade, Serbia; 4 HIV/AIDS Unit, Institute for Infectious and Tropical Diseases, School of Medicine, University of Belgrade, Belgrade, Serbia

## Abstract

**Aim:**

To compare four cardiovascular disease (CVD) risk models and to assess the prevalence of eligibility for lipid lowering therapy according to the 2013 American College of Cardiology/American Heart Association (ACC/AHA) guidelines, European AIDS Clinical Society Guidelines (EACS), and European Society of Cardiology and the European Atherosclerosis Society (ESC/EAS) guidelines for CVD prevention in HIV infected patients on antiretroviral therapy.

**Methods:**

We performed a cross-sectional analysis of 254 consecutive HIV infected patients aged 40 to 79 years who received antiretroviral therapy for at least 12 months. The patients were examined at the HIV-treatment centers in Belgrade and Zagreb in the period February-April 2011. We compared the following four CVD risk models: the Framingham risk score (FRS), European Systematic Coronary Risk Evaluation Score (SCORE), the Data Collection on Adverse Effects of Anti-HIV Drugs study (DAD), and the Pooled Cohort Atherosclerotic CVD risk (ASCVD) equations.

**Results:**

The prevalence of current smoking was 42.9%, hypertension 31.5%, and hypercholesterolemia (>6.2 mmol/L) 35.4%; 33.1% persons were overweight, 11.8% were obese, and 30.3% had metabolic syndrome. A high 5-year DAD CVD risk score (>5%) had substantial agreement with the elevated (≥7.5%) 10-year ASCVD risk equation score (kappa = 0.63). 21.3% persons were eligible for statin therapy according to EACS (95% confidence intervals [CI], 16.3% to 27.4%), 25.6% according to ESC/EAS (95% CI, 20.2% to 31.9%), and 37.9% according to ACC/AHA guidelines (95% CI, 31.6 to 44.6%).

**Conclusion:**

In our sample, agreement between the high DAD CVD risk score and other CVD high risk scores was not very good. The ACC/AHA guidelines would recommend statins more often than ESC/EAS and EACS guidelines. Current recommendations on treatment of dyslipidemia should be applied with caution in the HIV infected population.

Observational studies found higher rates of myocardial infarction and cerebrovascular events in HIV infected than in uninfected persons (1-6). Hence, prevention of cardiovascular disease (CVD) in HIV infected patients should be an integral part of current clinical practice. In routine HIV clinical care in developed countries it is recommended to calculate the CVD risk using prediction models (7-9). Preventable and modifiable predisposing factors for CVD should be identified, and lifestyle and pharmacological interventions should be undertaken.

CVD prevention in HIV-infected persons is mainly based on recommendations for the HIV uninfected population. The American College of Cardiology/American Heart Association (ACC/AHA) published the 2013 Guideline on the Assessment of Cardiovascular Risk (10) and the European Society of Cardiology and European Atherosclerosis Society (ESC/EAS) published one of the major European guidelines (11). The European AIDS Clinical Society (EACS) addresses many complications of HIV disease, including recommendations for lipid lowering therapy (7). All guidelines use different models for assessing cardiovascular risk. EACS recommends the Framingham Risk Scoring (FRS), while ESC/EAS recommends the European Systematic Coronary Risk Evaluation score (SCORE). The FRS has been widely used for estimation of coronary heart disease (angina, myocardial infarction, and coronary death), hard coronary events (myocardial infarction and coronary deaths), stroke, and global CVD (including CVD deaths, coronary disease, transient ischemic attack, and stroke) (12). SCORE estimates the 10-year risk of a first fatal atherosclerotic event (eg, myocardial infarction, stroke, aortic aneurysm), and calibrated versions exist to adjust for different death rates in European countries (13). These estimates have an important role in identifying high risk patients and in recommending lipid lowering therapy (7,11,14). ACC/AHA recommends the Pooled Cohort Equations for atherosclerotic cardiovascular disease (ASCVD) risk to evaluate the need for treatment of blood cholesterol levels in the non-HIV infected population (10).

CVD risk estimation formulas are primarily intended to assist physicians in identifying high risk healthy persons older than 40 years with no signs of clinical atherosclerotic disease (7,10,11,14). Patients with diabetes are generally recommended more intensive interventions and considered at a higher risk for CVD (11,14). Cardiovascular risk models designed for the HIV infected population have also been developed, the most widely known is the Data Collection on Adverse Effects of Anti-HIV Drugs Study (DAD) risk equation, which also includes HIV-specific variables such as duration of indinavir or lopinavir use and current use of indinavir, lopinavir, or abacavir (15). However, the follow-up in the DAD Study is still relatively short and the DAD risk equation has yet not been formally recommended for CVD risk assessment in routine clinical care for HIV-infected persons.

Southeastern European countries such as Bulgaria, Croatia, Hungary, Romania, and Serbia have high rates of age-standardized mortality from cardiovascular disease, (ie, ischemic heart disease and cerebrovascular disease) (16,17). The aim of our study was to analyze the agreement of the high DAD CVD score with other CVD scores (CVD-FRS, SCORE, ASCVD) developed for the non-HIV infected population in HIV infected patients receiving antiretroviral therapy (ART) in Croatia and Serbia. We also examined the prevalence of eligibility for lipid lowering therapy according to the 2013 ACC/AHA guidelines, EACS, and European ESC/EAS guidelines for cardiovascular disease prevention.

## Methods

### Settings

The study was conducted at the University Hospital for Infectious Diseases, Zagreb, Croatia and the HIV/AIDS Unit, Institute for Infectious and Tropical Diseases, School of Medicine, University of Belgrade, Belgrade, Serbia, in the period February-April 2011. Both Croatia and Serbia have a low-level HIV epidemic (18,19). Croatia has a centralized system of care for HIV and all patients are treated in one center. The number of patients treated per one calendar year in the period 2011-2013 ranged from 650 to 780. In Serbia, there are currently four HIV treatment centers. The Institute for Infectious and Tropical Diseases, School of Medicine, University of Belgrade was established in 1985 and is by far the largest one. In the period 2011-2013, it treated 1000 to 1200 people living with HIV per year. In Croatia and Serbia health care insurance is universal and free of charge, however, the number of approved antiretrovirals is limited, and older, less lipid friendly antiretrovirals, are still being used (20,21).

### Study population

We collected data on risk factors for CVD for all consecutive HIV infected patients seen at a scheduled visit for routine follow-up in the two centers. We included persons 40 to 79 years old in whom lipid measurements were done and who had at least 12 months of antiretroviral therapy (ART). We excluded pregnant women and patients with acute illness or exacerbation of a chronic illness at the time of evaluation. A total of 355 persons were seen at the treatment center in Zagreb, 132 of whom met our eligibility criteria. At the treatment center in Belgrade 712 persons were examined, 122 of whom met our eligibility criteria.

A standardized data collection form was developed which included information on sociodemographic characteristics, HIV disease characteristics (current CD4 cell count, HIV-1 viral load, past and current history of ART), known risk factors for CVD (family history of premature CVD, smoking, hypertension, age, sex), lipids (total cholesterol, HDL-cholesterol, LDL-cholesterol and triglyceride), blood glucose measurements, and hepatitis B and C coinfection. The study was approved by the Ethics Committee of the University Hospital for Infectious Diseases, Zagreb, Croatia and the Clinical Center of Serbia Ethics Committee.

### Definitions

CVD was considered present if a history of any of the following conditions existed on inclusion into the study: myocardial infarction, stable/unstable angina, invasive coronary artery procedure, carotid artery disease (symptomatic [eg, transient ischemic attack or stroke] or >50 percent stenosis on angiography or ultrasound), stroke, peripheral artery disease, abdominal aortic aneurysm or other forms of clinical atherosclerotic disease (eg, renal artery disease). Family history of premature cardiovascular events was considered present if the event occurred in a male first-degree relative <55 years or female first-degree relative <65 years. Diabetes mellitus was defined if at least two fasting plasma glucose levels were ≥7.0 mmol/L, casual plasma glucose >11.1 mmol/L, or if patient had a history of diabetes treatment.

Arterial hypertension was defined as systolic blood pressure >140 mm Hg and/or diastolic blood pressure >90 mm Hg (>135/85 mm Hg in diabetic patients) or taking antihypertensive drugs. Blood pressure was measured by the attending nurse using mercury sphygmomanometers. The patient was in a seated position; his or her arm was supported and flexed at the level of the heart. The stethoscope's bell was lightly pressed over the brachial artery just below the cuff's edge. The first knocking sound (Korotkoff) indicated the patient's systolic pressure and the disappearance of the knocking sound indicated diastolic pressure.

Dyslipidemia was considered present if total cholesterol >6.2 mmol/L, HDL-cholesterol <1.03, or fasting triglycerides >2.26 mmol/L. We used the modified updated National Cholesterol Education Program criteria for the definition of metabolic syndrome: triglycerides of at least 1.7 mmol/L, HDL of 1.0 mmol/L or less in men and of 1.3 mmol/L or less in women, hypertension (systolic blood pressure >130 mm Hg or diastolic blood pressure >85 mm Hg), or use of antihypertensive medications and a glucose level at least 5.6 mmol/L or the diagnosis of diabetes. Body mass index was used as a surrogate for waist circumference (22).

Ten-year risk for CVD was calculated using the Framingham equation (23) available as a web-based tool (*http://hivpv.org/Home/Tools*). The score was categorized as follows: <10% – low risk; 10%-20% – moderate risk; ≥20% – high risk. We also calculated the SCORE, which evaluates the 10-year risk of the first fatal atherosclerotic event (for example, myocardial infarction, stroke, and aortic aneurysm). We used the SCORE equation for high-risk countries, since the guidelines consider Croatia and Serbia as high-risk countries (13). Persons were considered at low risk if the score was <1%, at moderate risk if score was 1%-5%, and at high risk if the score was ≥5%. We applied the DAD risk equation exactly as published (15); a 5-year CVD risk >5% was considered high. The Pooled Cohort equation for ASCVD was calculated according to instructions in the 2013 ACC/AHA Guideline on the Treatment of Blood Cholesterol (10). The information required to estimate ASCVD risk includes age, sex, race, total cholesterol, HDL-cholesterol, systolic blood pressure, antihypertensive drug use, diabetes, and smoking status. The score estimates a 10-year risk for ASCVD for men and women 40 to 79 years of age. The analysis of agreement of high risk scores derived from the different CVD risk models included 213 individuals without known CVD events, without diabetes, and with total serum cholesterol ≤8 mmol/L and LDL-cholesterol ≤4.9 mmol/L.

Eligibility for lipid lowering therapy was assessed in 211 persons not receiving lipid lowering agents. We used three different guidelines; EACS, ESC/EAS, and the ACC/AHA guidelines. The current EACS guidelines use the CVD-FRS for defining eligibility for lipid lowering therapy. Therapy is recommended in patients with established CVD, diabetes, or 10-year CVD-FRS≥20% (7). We considered persons eligible for lipid lowering therapy according to the ESC/EAS guidelines if they already had a CVD event, diabetes, or total cholesterol ≥8 mmol/L (11). Individuals were also considered to be eligible if the calculated SCORE value was between 5 and 10% and the LDL-cholesterol was ≥2.5 mmol/L or the SCORE value was ≥10% and LDL-cholesterol was ≥1.8 mmol/L (11). Individuals in whom therapy is “considered” by the ESC/EAS guidelines were classified in the “not recommended” group. According to the 2013 ACC/AHA guidelines, individuals with the following conditions are eligible for lipid-lowering therapy: 1) presence of CVD, 2) primary elevations of LDL-C≥4.9 mmol/L, 3) diabetes with LDL-C 1.8 to 4.9 mmol/L, and 4) an LDL-C level 1.8 to 4.9 mmol/L with an estimated 10-year ASCVD risk of ≥7.5% and without established CVD or diabetes (10).

### Statistical analysis

Continuous variables were presented as medians and the first to third quartile (Q1-Q3) range. Categorical variables were presented as frequencies with percentages. The Agresti- Coull method was used for calculating 95% confidence intervals (CI) for proportions. We evaluated agreement between different high cardiovascular risk scores in a subset of 213 non-diabetic persons without a history of cardiovascular events and total cholesterol ≤8 mmol/L and LDL-cholesterol ≤4.9 mmol/L. This agreement was evaluated using the following cut-offs: 10-year CVD-FRS≥20%, 10-year SCORE≥5%, 10-year ASCVD risk ≥7.5%, and DAD CVD score >5%. Agreement between these categorized cardiovascular risk scores was assessed by observed agreement, agreement for higher and lower scores, and the Cohen’s kappa (ĸ) statistics. The delta method suggested by Mackinnon (24) was used for calculating the 95% CI for agreement for higher and lower scores. Patients who already used statins were not evaluated for the eligibilty for lipid lowering therapy (n = 43). Agreement for the eligibility for lipid lowering therapy by the EACS, ESC/EAS, and ACC/AHA guidelines was also assessed by ĸ statistics. The level of agreement was considered poor if ĸ = 0.20, fair if ĸ = 0.21-0.40, moderate if ĸ = 0.41-0.60, substantial if ĸ = 0.61-0.80, and very good if ĸ>0.80. All statistical analyses were done using SAS software, version 9.3. (SAS Institute, Cary, NC, USA).

## Results

A total of 254 patients (122 from Serbia and 132 from Croatia) were included in the study. (Table 1). Of 254 persons, 193 (76%) were men. Of 61 women, 43 (70.5%) were from Belgrade. Women were younger than men (median 47 vs 50 years). All study participants were Caucasians.

**Table 1:** Major HIV-disease characteristics of 254 HIV infected persons from Croatia and Serbia*

Characteristics	Total (N = 254)	Serbia (N = 122)	Croatia (N = 132)
Mode of transmission			
Men who have sex with men	75 (29.5)	11 (9.0)	64 (48.5)
Sex between men and women	94 (37.0)	41 (33.6)	53 (40.2)
Intravenous drug use	22 (8.7)	15 (12.3)	7 (5.3)
Other/unknown	63 (24.8)	55 (45.1)	8 (6.1)
Current CD4 cell count, mm^3^	484 (311-661)	452 (274-674)	507.5 (365-658)
Current viral load^†^			
HIV-1 RNA<200 copies/mL	242 (97.2)	115 (98.3)	127 (96.2)
HIV-1 RNA≥200 copies/mL	7 (2.8)	2 (1.7)	5 (3.8)
Current use of abacavir	79 (64.8)	138 (54.3)	59 (44.7)
Current use of lopinavir/ritonavir	94 (37.0)	47 (38.5)	47 (35.6)
Has antibody to hepatitis C virus	30 (11.8)	16 (13.1)	14 (10.6)
Hepatitis B surface antigen positive	19 (7.5)	12 (9.8)	7 (5.3)

*Values are n (%) or medians (Q1-Q3).

†5 patients from Serbia had no viral load measurement.

**Figure 1:**
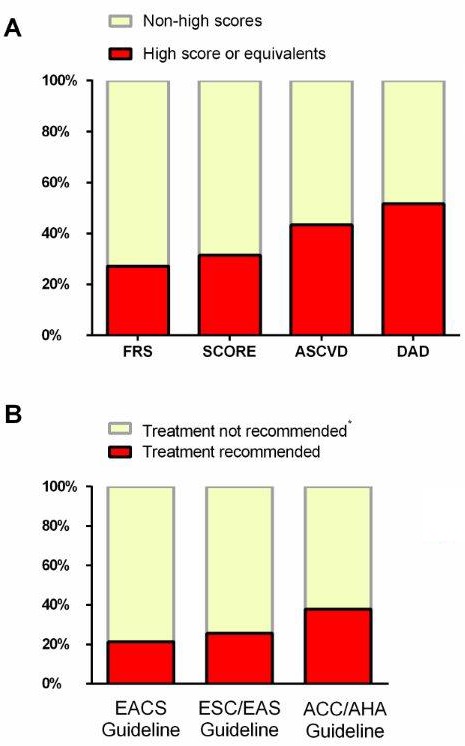
Prevalence of high cardiovascular disease risk scores or risk equivalents (FRS≥20%, SCORE≥5%, ASCVD≥7.5% and DAD>5%) according to different algorithms (**A**) in 254 participants and treatment recommendations for statin use (n = 211), based on the EACS, ESC/EAS, and the 2013 ACC/AHA guidelines (**B**). Patients with diabetes and clinical events were considered at high risk in the FRS and SCORE model. Patients with serum cholesterol >8 mmol/L were also in the high risk category in the SCORE model. All patients in the four major statin benefit groups were considered to have a high CVD risk. Patients who already used statins were not evaluated for eligibility for lipid lowering therapy (n = 43). *Includes participants in whom statin treatment was considered. FRS – 10-year Framingham Risk Score; SCORE – 10-year European Systematic Coronary Risk Evaluation score; DAD – 5-year Data Collection on Adverse Effects of Anti-HIV Drugs Study CVD score; ASCVD – 10-year atherosclerotic cardiovascular disease risk equation; EACS – European AIDS Clinical Society; ESC/EAS – European Society of Cardiology/European Atherosclerotic Society; ACC/AHA – American College of Cardiology/American Heart Association; CVD – cardiovascular disease.

The prevalence of high CVD scores or risk equivalents was noticeable and it ranged from 27.2% (69 of 254) for the CVD-FRS to 51.6% for the DAD score (131 of 254) (Table 2, Figure 1). Of note, 58.3% of persons had a CVD-FRS>10%, 71.5% of persons had a SCORE>1%, and 98.4% had a DAD score >1%. Current use of abacavir and the use of protease inhibitors contributed significantly to the high DAD CVD risk score (Table 2). Of 254 persons, 84 (33.1%) were overweight (body mass index, 25-29.9) and 30 (11.8%) were obese. A clinical CVD event was present in 8 persons (myocardial infarction, 6; stroke, 1; and coronary angiography with stenting, 1) and diabetes in 10.

**Table 2:** Prevalence of major cardiovascular risk factors and antiretroviral therapy by different high cardiovascular disease risk scores*^†^

	Overall (n = 254)	FRS≥20^‡^ (n = 69)	SCORE≥5^§^ (n = 80)	ASCVD≥7.5^║^ (n = 110)	DAD>5^‡^ (n = 131)
Age, years	49 (44-55)	57 (52-64)	57 (52-64)	55 (49-61)	54 (49-60)
Sex, male	193 (76.0)	65 (94.2)	72 (90.0)	95 (86.4)	114 (87.0)
Hypertension >140/90 or >135/85 mm Hg if diabetic	80 (31.5)	38 (55.1)	38 (47.5)	49 (44.5)	54 (41.2)
Diabetes mellitus	10 (3.9)	10 (14.5)	10 (12.5)	10 (9.1)	10 (7.6)
Current smoker	109 (42.9)	40 (58.0)	39 (48.8)	59 (53.6)	75 (57.3)
Ex-smoker	78 (30.7)	13 (18.8)	19 (23.8)	24 (21.8)	35 (26.7)
Cholesterol >6.2 mmol/L	90 (35.4)	29 (42.0)	41 (51.3)	55 (50.0)	60 (45.8)
HDL-C<1.03 mmol/L for men and <1.3 women	63 (24.8)	15 (21.7)	11 (13.8)	27 (24.5)	29 (22.1)
Triglycerides >2.26 mmol/L	96 (37.8)	34 (49.3)	33 (41.3)	61 (46.4)	61 (46.6)
Dyslipidemia^¶^	164 (64.6)	48 (69.6)	55 (68.8)	82 (74.6)	84 (71.8)
Family history of cardiovascular disease	20 (7.9)	3 (4.3)	3 (3.8)	5 (4.5)	7 (5.3)
Metabolic syndrome	77 (30.3)	30 (43.5)	29 (36.3)	41 (37.3)	47 (35.9)
Body mass index (kg/m^2^)	24.5 (22.3-27.3)	25.1 (23.5-27.8)	25.1 (23.5-27.8)	25.1 (22.9-28)	24.6 (22.8-27.6)
Current use of protease inhibitors	145 (57.1)	36 (52.2)	42 (52.2)	64 (58.2)	89 (67.9)
Current use of abacavir	138 (54.3)	43 (62.3)	50 (62.5)	68 (61.8)	91 (69.5)
Duration of protease inhibitor use, years	2 (0-6)	3 (1-5.6)	3 (1-5.6)	2.5 (0.9-6)	4 (1.1-6)
Duration of antiretroviral therapy, years	6 (3.5-10.4)	6 (4-10.1)	6 (4-10.1)	6 (3.6-10.6)	6.5 (4.1-11)
Current use of lipid lowering agents	43 (16.9)	24 (34.8)	24 (30.0)	30 (27.3)	33 (25.2)
Current use of antihypertensive medications	49 (19.3)	24 (34.8)	22 (27.5)	31 (28.2)	35 (26.7)

*FRS – 10-year Framingham Risk Score; SCORE – 10-year European Systematic Coronary Risk Evaluation score; DAD – 5-year Data Collection on Adverse Effects of Anti-HIV Drugs Study score; ASCVD – 10-year Atherosclerotic Cardiovascular Disease Risk Equation. HDL-C – high-density cholesterol.

†Values are n (%) or medians (Q1-Q3).

‡Also included patients with diabetes and clinical events.

§Also included patients with diabetes, clinical events and serum cholesterol >8 mmol/L.

║All patients in the four major statin benefit groups were considered to have an elevated CVD risk.

¶Dyslipidemia, any of the following: cholesterol >6.2 mmol/L or C<1.03 mmol/L for men and <1.3 women or triglycerides >2.26 mmol/L.

We evaluated agreement between the high 5-year CVD DAD score and the 10-year CVD-FRS, 10-year CVD SCORE, and 10-year ASCVD risk score in 213 non-diabetic persons without a history of cardiovascular events and serum cholesterol ≤8 mmol/L and serum LDL-cholesterol <4.9 mmol/L (Table 3). The agreement between the 5-year CVD DAD score and 10-year ASCVD risk score was better (ĸ = 0.63) than between the DAD score and CVD-FRS or SCORE algorithms (ĸ = 0.47 for both) (Table 3).

**Table 3:** Agreement between the 5-year DAD high CVD score and other high cardiovascular risk scores in 213 individuals between 40 to 79 years of age*^†‡§^

Cardiovascular risk scores	5-year DAD CVD score		Observed agreement	Agreement for higher scores	Agreement for lower scores	Kappa
≤5%	>5%
10-year CVD-FRS						
<20%	114	52	0.75 (0.68-0.80)	0.63 (0.53-0.72)	0.81 (0.76-0.86)	0.47 (0.36-0.57)
≥20%	2	45				
10-year SCORE						
<5%	114	52	0.75 (0.68-0.80)	0.63 (0.53-0.72)	0.81 (0.76-0.86)	0.47 (0.36-0.57)
≥5%	2	45				
10-year ASCVD						
<7.5%	110	32	0.82 (0.76-0.87)	0.77 (0.70-0.84)	0.85 (0.81-0.90)	0.63 (0.53-0.73)
≥7.5%	6	65				

*CVD – cardiovascular disease; FRS – Framingham Risk Score; ASCVD – atherosclerotic cardiovascular disease; SCORE – European System for Cardiac Operative Risk Evaluation.

†Values are frequencies or proportions with 95% confidence intervals in parenthesis.

‡Analysis included individuals without a prior CVD event, with total serum cholesterol ≤8 mmol/L, LDL-cholesterol ≤4.9 mmol/L, and those who did not have diabetes.

§Observed agreement = (a + d)/N; agreement for higher scores = (2*d)/(N-a + d); agreement for lower scores = (2*a)/(N+a-d); the letters denote cells in a 2 × 2 table (a, upper left; b, upper right; c, lower left; d, lower right; N = 213).

EACS guidelines would recommend lipid lowering therapy to 21.3% of persons (95% CI, 16.3% to 27.4%; n = 211); ESC/EAS guidelines to 25.6% (95% CI, 20.2% to 31.9%; n = 211); and ACC/AHA guidelines to 37.9% (95% CI, 31.6% to 44.6%; n = 211) (Figure 1). The use of ACC/AHA guidelines would increase the number of persons eligible for lipid lowering therapy by 77.8% (95% CI, 63.6%-87.6%) compared to the EACS guidelines that use the CVD-FRS score. The agreement between the guidelines in terms of indication for lipid lowering therapy was ĸ = 0.61, 95% CI, 0.51-0.72 (ACC/AHA vs EACS); ĸ = 0.71, 95% CI, 0.61-0.81 (ACC/AHA vs ESC/EAS); and ĸ = 0.71, 95% CI, 0.60-0.82 (EACS vs ESC/EAS). The use of abacavir in persons with high CVD risks was quite frequent ( ~ 60 to 70%) and the use of lopinavir/ritonavir was ~ 30% (Table 2).

## Discussion

We found a relatively high number of patients who needed interventions to reduce their cardiovascular disease risks. About one fifth to more than one third of patients, depending on the risk equation used, needed intensive lifestyle modifications (cessation of smoking, diet, or weight loss) and lipid lowering therapy. The agreement between the high DAD CVD scores and the elevated ASCVD scores was substantial (ĸ = 0.63), however, the agreement between high DAD and high CVD-FRS, and the high DAD with high SCORE algorithm was only moderate (ĸ = 0.47). Hence, none of the agreements were very good (ĸ>0.80). Agreement for lower CVD scores was better than for higher scores (Table 3).

Although high CVD scores are used to make clinical decisions about initiating statin therapy, concordances of those high score categories derived from different algorithms have not, to our knowledge, been reported in the HIV infected population. De Socio et al reported data on cardiovascular risk profiles using different risk models in 2007 (25) and 2008 (26). They found more men in the high Framingham coronary heart disease risk strata (>20%) than in the high SCORE algorithm group (>5%) (26). However, it is difficult to compare different studies because they include different populations and use different risk algorithms. For example, in the study by De Socio et al the mean age was 43 years and the mean SCORE percentage was 1.1. In our study, the mean age was 50.3 years and the mean SCORE percentage was 3.3. Also, the study by De Socio et al had been done before the DAD algorithm was developed.

CVD prediction algorithms have been developed with the purpose of identifying persons without known CVD who are at high risk of a cardiovascular event. They aim to aid physicians to decide when and how to use lipid lowering drugs. However, there is currently no consensus on which CVD risk algorithm should be used in the HIV infected population. The EACS guidelines recommend statin therapy, in addition to lifestyle modifications, when the 10-years CVD-FRS is ≥20% (7). However, it has been reported that the FRS underestimates the occurrence of stroke (27) and myocardial infarction (28) in HIV infected patients. For the treatment of high blood cholesterol in the non-HIV infected population the use of the ASCVD algorithm has been recently suggested (10).

In our population of HIV infected patients without previous use of lipid lowering medications, we found that the recent ACC/AHA guideline would recommend statin therapy to 38% of persons. The application of the 2013 ACC/AHA guidelines that use the ASCVD score compared to the EACS guidelines that use the CVD-FRS would increase the number of eligible patients for lipid lowering therapy by 78%. The new cholesterol guidelines have also been recently compared to other guidelines in the non-HIV infected population (29). An analysis of 4209 individuals not receiving statins in the prospective Rotterdam cohort aged >55 years found that the new cholesterol guidelines would have recommended statins to 96% of men and 66% of women (29). Of note, 66.1% of men and 39.1% of women were included in the “treatment recommended” category by the ESC/EAS guidelines (29). In a recent study, Zanni et al compared the 2013 ACC/AHA guidelines with the 2004 Adult treatment Panel III Cholesterol guidelines in HIV infected patients with or without subclinical high risk coronary plaque (30). Similarly to our findings, the 2013 ACC/AHA cholesterol guidelines recommended statin therapy to a higher percentage of patients than 2004 guidelines (30).

The prevalence of cardiovascular risk factors in our sample of HIV infected patients from Croatia and Serbia was high: hypertension (31.5%), smoking (41.9%), past smoking (30.7%), dyslipidemia (64.6%), and overweight or obesity (44.9%). The prevalence of hypertension and dyslipidemia was higher or similar to recent studies from the USA (31), Switzerland (32,33), Italy (34), and Germany (35).

The increased risk of CVD among HIV infected people is associated with traditional factors such as age, male sex, hypertension, smoking, diabetes mellitus, pre-existing CVD, and hypercholesterolemia as in the general population (36). However, HIV infection itself is a CVD risk factor for subclinical atherosclerosis and clinical cardiovascular events (37-40). A low CD4+ cell count was found to be a risk factor for cardiovascular disease events (41,42). Certain drugs used for HIV treatment, eg, lopinavir/ritonavir, indinavir/ritonavir, amprenavir, and fosamprenavir have also been associated with an increased risk for myocardial infarction (43,44). There is a considerable controversy about the association of abacavir with CVD (44,45), and guidelines caution about abacavir use in patients with a high CVD risks (7). In our study, about 60% of patients with a high CVD risk were using abacavir. Given the unclear relationship between abacavir and CVD events (44,45), it seems prudent to avoid using abacavir in patients with high cardiovascular risk if other alternatives exist. However, in Croatia and Serbia abacavir is frequently used due to the unavailability of tenofovir in Serbia and higher cost of tenofovir-based formulations compared to abacavir formulations in Croatia.

Our study has several limitations. Due to cross-sectional design, there is a lack of data on the changes in CVD risk over time. The analysis included only patients between 40 and 79 years old, so the frequency of CVD risks might be higher than in the studies that included younger age groups. However, the age range we used is the age range in which different CVD risk equations have been primarily developed and validated. Different CVD scores can also be compared as interval variables with graphs such as the Bland-Altman graph (46). However, as interventions to reduce CVD risks are usually made by some cutoffs we decided to compare CVD risk models by the cutoff at which more intensive interventions are recommended. We were also unable to make a more detailed analysis of whether the targets for lipids or blood pressure were reached in patients using lipid lowering or blood pressure lowering therapy. Our study sample was not large and included only Caucasian individuals. Finally, our point prevalences of different cardiovascular risk factors should be interpreted with caution because of the convenience sampling method used.

Our data suggest that clinicians have to make decisions on lipid lowering therapy using inconsistent recommendations. Until more data with clinical outcomes becomes available, physicians need to decide which of the different available algorithms is best for predicting CVD events in their patients. We would prefer to use an algorithm and recommendations specifically developed for the HIV population. It also has to be remembered that the interpretation of a particular CVD risk algorithm should always include careful clinical assessment and judgment.

## 

Accepted: January 20, 2015
